# Evaluation of estimated genetic values and their application to genome-wide investigation of systolic blood pressure

**DOI:** 10.1186/1753-6561-8-S1-S66

**Published:** 2014-06-17

**Authors:** Ellen E Quillen, V Saroja Voruganti, Geetha Chittoor, Rohina Rubicz, Juan M Peralta, Marcio AA Almeida, Jack W Kent, Vincent P Diego, Thomas D Dyer, Anthony G Comuzzie, Harald HH Göring, Ravindranath Duggirala, Laura Almasy, John Blangero

**Affiliations:** 1 Department of Genetics, Texas Biomedical Research Institute, PO Box 760549, San Antonio, TX 78245, USA; 2 Centre for Genetic Origins of Health and Disease, University of Western Australia, 35 Stirling Hightway, Crawley, Western Australia, 6009, Australia

## Abstract

The concept of breeding values, an individual's phenotypic deviation from the population mean as a result of the sum of the average effects of the genes they carry, is of great importance in livestock, aquaculture, and cash crop industries where emphasis is placed on an individual's potential to pass desirable phenotypes on to the next generation. As breeding or genetic values (as referred to here) cannot be measured directly, estimated genetic values (EGVs) are based on an individual's own phenotype, phenotype information from relatives, and, increasingly, genetic data. Because EGVs represent additive genetic variation, calculating EGVs in an extended human pedigree is expected to provide a more refined phenotype for genetic analyses. To test the utility of EGVs in genome-wide association, EGVs were calculated for 847 members of 20 extended Mexican American families based on 100 replicates of simulated systolic blood pressure. Calculations were performed in GAUSS to solve a variation on the standard Best Linear Unbiased Predictor (BLUP) mixed model equation with age, sex, and the first 3 principal components of sample-wide genetic variability as fixed effects and the EGV as a random effect distributed around the relationship matrix. Three methods of calculating kinship were considered: expected kinship from pedigree relationships, empirical kinship from common variants, and empirical kinship from both rare and common variants. Genome-wide association analysis was conducted on simulated phenotypes and EGVs using the additive measured genotype approach in the SOLAR software package. The EGV-based approach showed only minimal improvement in power to detect causative loci.

## Background

Given increasing evidence that the majority of variation in common, complex traits is the result of a large number of individual variants with small effects, refining phenotypes to minimize the environmental component is one possible approach to increasing power to detect these variants. This work extends a common concept in plant and animal breeding, the estimated breeding value, to calculate estimated genetic values (EGVs) in human pedigrees. The breeding value is the deviation of an individual's phenotype from the population mean as a result of the sum of the average effects of the genes they carry. There are several methods for estimating breeding values, with the Best Linear Unbiased Prediction (BLUP) used most frequently. In its most basic form, BLUP accounts for additive genetic and environmental covariances among relatives based on known pedigree structure. Several extensions of BLUP have been developed to calculate the genomic estimated breeding values, which are derived directly from molecular genetic information and are commonly used for genomic selection in plant and animal breeding programs [1,2]. The accuracy of and variation in EGVs are increased when phenotype heritability estimates are higher and by the inclusion of additional information from relatives, so the method is most useful in extended pedigrees. Because EGVs represent predominantly additive genetic variance (some additional environmental variance may be included where it mimics relatedness), the use of EGVs in place of standard phenotypes will increase heritability and may also increase power to detect variants of smaller effect. Although high heritability does not guarantee the identification of causal variants [3], it is one possibility out of a variety of methods to increase the identification of variants that frequently fall below the significance threshold in genome-wide association (GWA) studies. Zabaneh and Mackay [4] examined the suitability of using pedigree-based EGVs in genome-wide linkage analysis, and found that this method improves power to detect quantitative trait loci. However, because EGVs are a product of genetic similarities of individuals in the sample, the use of empirically derived relationship matrices in calculating EGVs should increase power to localize genetic factors in genome-wide association (GWA) studies, an observation that has driven the use of relationship matrices in artificial selection [[5] and others].

## Methods

### Sample description

To determine the suitability of EGVs for human quantitative traits, the simulated visit 1 systolic blood pressure (SBP) values from the Genetic Analysis Workshop 18 (GAW18) data set were considered [6]. R princomp [7] was used to perform principal components analysis on the 117 unrelated individuals in the sample using a subset of 28,157 single-nucleotide polymorphisms (SNPs) selected for uniform coverage and low mutual linkage disequilibrium (LD) from the SNPs provided. The resulting principal component (PC) scores were projected on the full set of related individuals by assigning offspring the mean of parental scores. Using SOLAR [8], residual SBP values for each simulation were obtained from fitting a polygenic model incorporating age, sex, and the first three PCs as covariates. The value of these covariates remained the same across all simulations, but their effects on SBP varied. Expected relatedness (2Φ) based on the provided pedigree was calculated in SOLAR. Additionally, empirical relatedness was calculated in KING [9], based on either common variants (472,050 SNPs located on odd-numbered chromosomes) or all variants extracted from the sequence data on odd-numbered chromosomes. Of the 2 methods offered in KING, the robust method was selected as it employs a family-specific correction for substructure.

### EGV calculation

The residualized SBP values and relationship matrices, **R**, equal to 2Φ were used in calculating EGVs. Throughout, EGVs will be subscripted with the origin of the **R **matrix so that EGV_ped _is derived from the expected, pedigree-based matrix, EGV_snp _from the empirical SNP-based matrix, and EGV_seq _from the full sequence-based matrix. EGVs were calculated from a variation of the standard BLUP estimation of breeding values using a custom script written in GAUSS (Aptech Systems, Inc.), which is available upon request. At the core of the calculation is the mixed model

$$EGV={\left(I+R^{-1}\frac{\sigma_{e}^{2}}{\sigma_{g}^{2}}\right)}^{-1}\left(y-X\beta \right)$$

where ***X ***is the matrix of fixed effects (sex, age, and the first 3 PCs) included as covariates in the polygenic analysis, *y *is the observed value of SBP, and *β *is the fixed-effects coefficient. In full, y-Xβ is the residual values derived from the variance component model discussed above. The additive genetic variance $\left(\sigma_{g}^{2}\right)$ and environmental variance $\left(\sigma_{e}^{2}\right)$ are also obtained from the polygenic model fit in SOLAR. Because the **R **matrices produced by KING are not positive, semidefinite--a requirement for solving the model--the nearest correlation matrix was calculated by the alternating projections procedure described by Higham [10] and implemented in GAUSS [11].

### Genome-wide association

GWA was performed in SOLAR for the raw SBP values, EGV_ped_, EGV_snp_, and EGV_seq_. Although EGVs can be estimated for unphenotyped individuals, sample size remained the same across all tests as the simulated data set has phenotype and genotype information for all 847 nonidentical individuals.

GWA was performed using the measured genotype association (MGA) test, which applies a likelihood ratio test to an additive model of allelic effects while including a covariance matrix of expected pairwise relatedness to control for kinship. The pairwise error variance matrix calculated in the GAUSS script was also incorporated into the MGA model such that

$$\Omega ={\text{σ}}_{p}^{2}\left(2\text{Φ}h^{2}+I\sigma_{e}^{2}+E\varepsilon \right)$$

where *Ω *is the phenotypic covariance matrix, Φ is the expected kinship matrix, *h^2 ^*is narrow-sense heritability, **I **is the identity matrix, $\sigma_{p}^{2}$ and $\sigma_{e}^{2}$ are phenotypic and environmental variance components, **E **is the error variance matrix, and ε is the corresponding error parameter. Under the MGA test, the log-likelihood of the null model with no SNP effect is compared to the log-likelihood of the alternative model with an estimated SNP effect. The likelihood ratio test, given as minus twice the difference of the null and alternative log-likelihoods, is distributed as a chi-square variate with 1 degree of freedom.

A major advantage of the simulated data is the ability to determine the precise false-discovery rate of the method. For each of the top 55 variants contributing to simulated SBP and all SNPs identified in the GWA analyses (*p *<5 × 10^−7^), pairwise correlations (R^2^) were calculated in SOLAR to assess the extent of local LD. An associated SNP was considered accurately identified if it fell within an LD block defined by R^2 ^≥0.2 surrounding a simulated causative gene; otherwise the associated SNP was deemed a false positive.

## Results

### Expected and empirical relatedness

The use of empirical relatedness measures is increasing in popularity as a means of resolving the variation around pedigree-based relatedness estimates and accounting for cryptic relatedness and inbreeding. These factors will increase mean relatedness in a population, as reflected in the larger SNP-based and sequence-based pairwise kinships relative to the pedigree-based values. In all cases, the heritability of the EGVs is approximately double the heritability of simulated SBP with covariates included. This is expected as the computation of EGVs removes much of the environmental variation seen in SBP. However, this also indicates that not all nongenetic variation has been removed.

### GWA results from EGVs

There is broad variation in the number of significantly associated SNPs (*p *<5 × 10^−8^) across the simulations and within or among methods. On average, EGV_snp _and EGV_seq _identified more SNPs than the raw SBP GWA, but the median number of associations was approximately the same (Table 1). The simulation was designed so that 15 SNPs in *MAP4 *comprise 7.79% of the variation in SBP, 14 SNPs in *NRF1 *4.67%, 16 SNPs in *TNN *3.87%, and 8 SNPs in *LEPR *2.06%. Additional genes had extremely small effects. Table 2 shows the number of simulations in which each expected gene was identified for each method. All methods reliably identified SNPs in *MAP4 *and neighboring gene *DNASE1L3*, with additional SNPs in *BTD *and *SUMF1 *also associated because of the extended LD and the large effect of *MAP4*. SNPs in *LEPR *were only identified in 4% to 7% of cases, with no method clearly outperforming the others. *MAP4 *and *LEPR *each contained a single SNP contributing more than 2% of the variance, which is likely why no SNPs in *NRF1 *or *TNN *were identified despite these genes contributing more to SBP in their entirety. Among the genes that explain less than 2% of the overall variance, none was identified by any method in more than 5% of trials.

**Table 1:** Description of GWA results for EGVs and SBP across simulations.

	SBP	EGV_ped_	EGV_snp_	EGV_seq_
Total number of significant (sig) SNPs in 100 simulations	166	134	191	273
Sig SNPs with minor allele frequency (MAF) <0.05	12.0%	5.2%	11.5%	6.6%
Sig SNPs with MAF <0.01	1.2%	0.7%	1.0%	0.7%
Mean number of sig SNPs	11.9	7.8	13.9	14.1
Median number of sig SNPs	8	2	8	8
stdev in number of sig SNPs	15.8	15.0	18.4	19.3
False-discovery rate (FDR)	7.7%	5.7%	6.7%	7.7%
FDR for SNPs not seen in SBP	-	52.1%	50.0%	40.4%
Smaller average *p *value for sig SNPs	-	30.0%	57.0%	48.0%
Simulations identifying more SNPs than SBP	-	14	39	36
Simulations identifying fewer SNPs than SBP	-	77	18	30
Simulations identifying same number of SNPs as SBP	-	9	43	34

For 100 replicates of simulated SBP, GWA was performed on the raw data, EGV_ped_, EGV_snp_, and EGV_seq_. The following table gives descriptive statistics for significant SNPs (*p *< 5 × 10^-^^8^) by method. The last five rows illustrate the performance of the EGV method relative to the raw SBP GWA.

**Table 2:** Significant results for GWA of EGVs and SBP by gene.

Gene	Chr	SBP	EGV_ped_	EGV_snp_	EGV_seq_
*LEPR*	*1*	7	4	6	4
*LRP8*	*1*	6	0	8	5
*NEXN*	*1*	1	1	2	1
*BTD*	*3*	16	16	22	20
*DNASE1L3*	*3*	100	95	100	99
*MAP4*	*3*	100	95	100	99
*SUMF1*	*3*	33	14	34	39
*MTRR*	*5*	3	0	3	4
*RHOD*	*11*	2	0	1	1
*TCIRG1*	*11*	2	1	1	1
*CYP1A2*	*15*	0	0	1	0
*C1QBP*	*17*	0	1	0	1
*KRT23*	*17*	0	1	0	1
*RAI1*	*17*	0	1	0	1
*SAT2*	*17*	0	0	1	1
*COL5A3*	*19*	0	1	1	0

For each gene contributing to simulated SBP, the table lists the number of replicates (out of 100) in which at least one significant association was found. Variants in 24 additional genes have small effects on SBP but were never detected and were omitted from the table. Due to extended linkage disequilibrium, more than one gene may be tagged by a single variant; in particular, the associations in *DNASE1L3 *are likely due to strong LD with major causative gene *MAP4*.

Of particular interest for complex traits like SBP is the effect of rare variants, a feature reflected in the design of the simulation with 10 of the top 55 causal variants present at minor allele frequency (MAF) less than 1% and 28 at less than 5%. In contrast, less than 12% of significantly associated SNPs identified by any method have a MAF ≤0.05 and only 1% have a MAF ≤0.01.

Overall, the false-discovery rate (FDR) is low, with FDR approximately stable across the raw SBP and EGV GWAs (see Table 1), and little evidence of inflation in lambda values. However, as this method aims to move contributing variants out of the suggestively associated "gray zone" without inflating type II error, the error rate was also calculated specifically for SNPs associated with the EGV values, but not the raw SBP values. These FDRs were greatly inflated, with approximately half of these associations representing false positives (see Table 1).

## Discussion

There are several measures of the utility of a novel method of GWA: strength of associations, absolute number of significant associations, number of genes or genomic regions identified, and the minimization of type I and type II error rates. Strength of association and number of significant associations will be strongly correlated and were expected to be the predominant avenue of improvement in the use of EGVs. Ideally, the use of EGVs would eliminate the influence of environmental variation and drive more causative SNPs from the "suggestive" into the "significant" association range of *p *values. Although the use of EGVs does increase heritability by removing environmental variation and capturing only additive genetic variation, it does not guarantee a sufficient increase in power to detect extremely rare variants or deal with phenotypes with a very large number of causative genes (e.g., height). Based on these simulations, where many alleles with a low MAF and small effect sizes contribute to SBP, this method does not substantially increase the power to detect rare variants. This method is most likely to improve power in studies of large pedigrees where individuals have many close relatives, which maximizes accuracy of EGV calculation, in phenotypes with significant but difficult-to-quantify environmental components that can be removed by the EGV method, and where the majority of genetic variation is a result of additive effects.

## Conclusions

The EGV_snp _and EGV_seq _methods, which employ empirical kinship estimates, slightly outperformed the standard MGA method based on the average number of truly causal SNPs identified. However, when judged on the basis of additional causal genes identified, the improvements are sporadic and fail to recognize genes of medium effect. Overall, the use of EGVs neither significantly increased nor decreased the ability to detect rare causal variants of small to modest effect.

## Competing interests

The authors declare that they have no competing interests.

## Authors' contributions

JB designed the overall study and assisted in statistical analyses. EEQ conducted the statistical analyses and drafted the manuscript; VSV, GC, and RR helped in statistical analyses and drafting the manuscript. JMP, MAAA, JWK, VPD, and TDD contributed additional data for the analyses. RD, HHHG, LA, and AGC contributed analytical oversight and comments. All authors approved the final manuscript.
